# Moving from subjectivity to standardization? The emerging role of artificial intelligence in the embryology laboratory

**DOI:** 10.1016/j.xfre.2026.04.011

**Published:** 2026-05-02

**Authors:** Rachel A. Martel, Jason Franasiak

**Affiliations:** a Reproductive Medicine Associates of New Jersey, Basking Ridge, New Jersey; b Division of REI, Department of OB/GYN, Thomas Jefferson University, Philadelphia, Pennsylvania

In recent years, artificial intelligence (AI) has become a powerful tool across multiple industries. Many AI applications have been developed for use in medicine; however, there is an extremely high bar for implementation and studies must prove effectiveness before use. The field of reproductive endocrinology offers multiple potential applications for AI automation and improvement, but there perhaps is no area that stands to benefit more than the embryology laboratory where precision, reproducibility, and objectivity are of utmost importance. Particular emphasis has been placed on embryo assessment given the clear correlation between embryo grade and embryo transfer outcomes. Thus, AI models that assess and grade embryo quality have been developed in an effort to decide which embryos are most fit for transfer.

This would seem an intuitive solution given the subjectivity associated with embryo assessment. The inherent subjectivity involved in embryo grading and the potential for AI to improve consistency has been demonstrated in several studies, including a recent prospective, double-blind study that concluded that a neural network outperformed embryologists in terms of consistency when evaluating and grading embryo images (1). In this study, embryologists had an average consistency of 52.14% when selecting embryos for biopsy and 57.68% when selecting embryos for cryopreservation. In contrast, the neural network significantly outperformed embryologists with a consistency of 83.92% for both tasks. Cronbachs α tests demonstrated significantly improved reliability in the neural network vs. the embryologists decision making with an α value of 1.0 vs. 0.6

Picking the best embryo is critically important and some evidence suggests that AI may do a better job at predicting which embryos go on to develop a fetal heart rate. A retrospective cohort study found that among day 3 and day 5 embryos selected for transfer, an AI-based scoring system significantly outperformed manual morphology assessment at predicting the presence of fetal heart tones at 28–36 days after embryo transfer (2). For day 3 and day 5 transfers, the area under the curve for fetal heartbeat discrimination was significantly higher for the AI scoring system (0.63 vs. 0.59 on day 3, 0.59 vs. 0.55 for day 5) and this remained significant when the analysis was restricted to women <35 or >35 years old.

It is known that embryologists gain accuracy with time and experience, which poses the question: can AI support tools have a role in embryologist training and development? Yet exactly how, or if, AI should be integrated into the embryology laboratory remains an open question. Whether used to improve standardization, decision support, or embryologist training we must think critically about how AI can measurably improve patient outcomes before implementation.

In this edition of *Fertility and Sterility Reports*, Berntsen et al (3) present their article entitled Embryologist Experience Affects Concordance with an AI Embryo Ranking Algorithm: Benefit of AI Assistance, which looks critically at when AI might be most useful in the embryology laboratory. The study systematically examines how embryologist experience level affects concordance with AI. They completed a retrospective analysis of 1,321 treatment cycles with 19,962 embryos at one urban, United States fertility center from February 2020–October 2023. Blastocysts were assessed on days 5 and 6 manually according to the Gardner Scale and by assigning a score using the iDAScore v2, an automated AI-based embryo scoring algorithm that generates a relative score from 1.0–9.9 correlating with the likelihood of development of a fetal heartbeat for each embryo. Embryo selection was considered concordant if the blastocyst selected manually for first transfer also was given the highest iDAScore value among the available euploid embryos. Manual evaluation was completed by embryologists of differing experience: Junior (0–3 years), Senior (3–5 years), and Senior+ (≥5 years). Their primary clinical outcome was the presence of fetal heart tones in the sixth week of gestation.

The investigators found an overall embryologist and iDAScore concordance rate of 58.6%, with concordance rate increasing with the experience level of the embryologist. Importantly, they found that Senior+ embryologists were significantly more likely to be concordant with the AI algorithm than Senior and Junior embryologists. Of note, the investigators reported that the concordance rate decreased with increasing numbers of usable embryos. Additionally, the presence of fetal heart tones in the sixth week of gestation increased with the experience level of the embryologist and with the number of usable embryos. In an adjusted analysis, they found that fetal heart tones in the sixth week of gestation were significantly more likely to be present in the concordant vs. discordant groups overall and when the analysis was limited to patients with 4–8 euploid embryos available for transfer. On the basis of these findings, they suggest that an AI algorithm may be a useful adjunct and/or training tool for junior embryologists gaining experience with grading embryos. They also posit that AI may be particularly useful for patients with an intermediate number of embryos (4–8), as these patients have a sufficient number of embryos to increase the complexity and subjectivity of embryo ranking, but not so many that their outcome is likely to be positive regardless of embryo assessment.

Berntsen et al (2) thus suggest key areas within the embryology laboratory that may benefit from targeted use of AI, acknowledging the inherent limitations of a retrospective, single center analysis and the inability to control for human factors that may affect embryologist performance. As they identify, these results may not be generalizable to a broader patient population, particularly populations where use of PGT-A testing is not universal. Others have similarly urged caution with applying models developed on a specific cohort of patients to the general population, and raised concerns as to whether large data sets can truly account for the specific differences between couples (4). It remains to be seen whether a model developed and trained on a set of embryos in one laboratory will translate well to others around the country or the world. Models also may not adapt well to different embryo imaging systems (1). However, it must be noted that the issue of generalizability also is present with regards to manual assessment. Embryologists are trained by their peers and by the embryos that they encounter in one specific laboratory which creates the potential for substantial variability in embryo grading between different clinics and laboratories. With proper validation, AI may provide an opportunity for standardization not only within, but between different laboratories. Thus, additional data and prospective studies are needed to address AI model performance across several different clinics and patient populations.

In additional to generalizability, investigators have raised concerns over the “black box” problem in AI, or the lack of transparency in how AI models produce their outputs. Many models are not designed to be interpretable or are simply too complex to be understood by a human. This creates several ethical and logistical challenges to AI implementation. Chief among them is the inability to explain embryo selection to patients resulting in compromised shared decision making and the risk that AI may unknowingly over-select for traits that increase implantation but also carry disadvantages with regard for future health or do not align with the values of the parents. There also is a potential inability to identify confounders, troubleshoot, and check for errors in real time. These concerns underscore the importance of using AI as an adjunctive rather than a replacement tool, with continued insight and oversight from embryologists. Interpretable AI models that break down the decision-making process into discrete stepwise scores or decision trees have been developed and may alleviate some of the concerns listed above (5). If, as Berntsen et al (3) suggest, AI may be a useful adjunct for junior embryologists, implementation will need to be performed with careful human oversight and preferably with interpretable models.

An additional limitation within the literature on AI embryo selection is the use of surrogate clinical endpoints rather than live birth. Few studies have examined whether increased implantation rates in the setting of AI use translate into clinically meaningful increases in live birth. Indeed, a prospective study in which 250 patients with multiple high-quality day 3 embryos were assigned to either AI or conventional morphological assessment (by patient preference) found improved implantation but not live birth rates in the AI group (5). Thus, while embryologist-AI concordance was associated with the presence of fetal heart tones in the study by Berntsen et al (3), it remains to be seen whether their conclusions are applicable to live birth either overall or on a per cycle basis. Ultimately, this is the most important clinical endpoint and evaluating the impact on live birth rates is crucial before implementing any new technology.

With these concerns in mind, future research is needed to address whether embryologist-AI concordance truly is an important performance benchmark. Prospective studies are needed to evaluate whether concordance with an AI model significantly improves live birth as well as implantation rates, and whether this changes according to embryologist experience level and number of embryos available for transfer. Additionally, multicenter studies are needed to evaluate model performance across multiple different patient populations and laboratories. Ultimately, AI clearly holds promise for enhancing consistency and supporting embryologist decision-making, but its integration into the embryology laboratory must be guided by prospective evidence that demonstrates validation across diverse settings and improvement in patient outcomes.

## CRediT Authorship Contribution Statement

**Rachel A. Martel:** Writing – original draft. **Jason Franasiak:** Conceptualization, Writing – review & editing.

## Declaration of Interests

R.A.M. has nothing to disclose. J.F. has nothing to disclose.
